# The Costs and Their Determinant of Cesarean Section and Vaginal Delivery: An Exploratory Study in Chongqing Municipality, China

**DOI:** 10.1155/2016/5685261

**Published:** 2016-11-22

**Authors:** Zhifei He, Zhaohui Cheng, Tailai Wu, Yan Zhou, Junguo Chen, Qian Fu, Zhanchun Feng

**Affiliations:** ^1^School of Medicine and Health Management, Tongji Medical College, Huazhong University of Science & Technology, No. 13 Hangkong Road, Wuhan, Hubei 430030, China; ^2^School of Politics and Public Administration, Southwest University of Political Science and Law, No. 301 Baosheng Road, Chongqing 401120, China; ^3^Institute of Higher Medical Education, Third Military Medical University, No. 35 Gaotanyan Road, Chongqing 401120, China

## Abstract

*Objectives*. This study aims to analyze the cesarean section (CS) rates and vaginal delivery rates in tertiary hospitals of China, explore the costs of two different deliveries, and examine the relative influencing factors of the costs in both CS and vaginal deliveries.* Methods*. 30,168 anonymized obstetric medical cases were selected from three sample tertiary hospitals in Chongqing Municipality from 2011 to 2013. Chi-square test was used to compare the distributions of CS and vaginal deliveries under different indicators. Mann–Whitney test and Kruskal-Wallis test were adopted to analyze the differences under different items. Multiple linear regression was used to determine the influencing factors of the costs of different delivery modes.* Results*. (1) The rates of CS were 69%, 65.5%, and 59.2% in the three sample tertiary hospitals in Chongqing from 2011 to 2013. (2) The costs and the length of stay of CS were greater than those of vaginal delivery, which had significant differences (*P* < 0.005). (3) The areas, length of stay, age, medical insurance, and modes of delivery were the influencing factors of both CS and vaginal delivery costs.* Discussion*. The high CS rates in China must be paid significant attention. The indicators of two modes of delivery should be regulated strictly. CS rate reduction and saving medical resources will be the benefits if vaginal delivery is chosen by pregnant women.

## 1. Introduction

Pregnancy is a normal and healthy state, to which most women aspire at some points in their lives, although it carries serious risks of death and disability [1]. Around the turn of current century, global estimates in 126 countries showed that, on average, 15% of births were delivered by CS; however, great variation exists among regions and countries [2]. Nearly over half a million of young women die because of complications arising from pregnancy and childbirth every year, and according to statistics, most of these deaths occur in the developing world [3].

**Figure 1:**
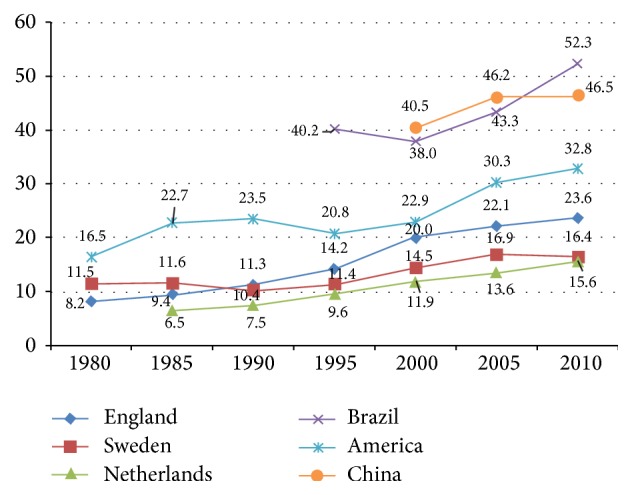
Trends in cesarean section (CS) rates (per 100 births), in selected countries, 1980–2010.

CS rates are increasingly epidemic nearly worldwide. In 1985, the World Health Organization (WHO) recommended that the optimal CS rates should not be higher than 10% to 15%, and this recommendation has become a reference up to this day [3]. The levels of 10%–15% were considered high but acceptable at the time. However, the average CS rates in the majority of developed regions (with the exception of Eastern Europe) currently exceed 20% [4, 5]. The reasons for the increasing CS rates are complicated, such as clinical indications, socioeconomic factors, previous CS, pregnant request with no medication, obstetric attitudes, and health care system [6]. According to previous literature, different countries have different CS rates (Figure 1). From 1980 to 2010, the CS rate in Sweden and Netherlands was less than 20%, whereas the CS rate in England was between 20% and 30%. In the United States, the CS rate was more than 30%, which is similar to that in China and Brazil. Meanwhile, the trends of CS rates in different countries increase every year [7].

In Figure 1, the horizontal axis represents the years from 1980 to 2010, while the vertical axis represents cesarean section (CS) rates (per 100 births). The different lines' color represents different countries. The trend of each line represents that, with the year changes, the CS rates increase.

In China, the CS rates increased from approximately 5% in the 1960s to approximately 20% in the late 1980s and the early 1990s [8]. Since the mid-1990s, the CS rates in the urban cities of China increased dramatically to 39.5% from 1998 to 2002 [9]. According to an official report by the WHO published in* The Lancet*, a global survey of WHO on pregnant and perinatal health showed that the CS rates in China were 46.2% during 2007-2008, which ranked first in Asia and ranked second in the world [10]. The CS without medical indications are unnecessary, and the number of unnecessary CS cases in 2008 in China was 1,976,606 [11]. Some reasons caused the high CS rate, such as the health care insurance. Chinese government has announced launching three different types of health care insurance in recent years: Urban Employee Basic Medical Insurance (UEBMI), Urban Resident Basic Medical Insurance (URBMI), and New Cooperative Medical Scheme (NCMS) [12], to target different population groups and provide universal coverage of health services with a focus on equity in health and health care utilization. In addition, previous literature has shown that these unnecessary CS have a negative effect on pregnant and neonatal health. Unnecessary CS not only increases the health risk of pregnant women [13, 14] but also results in unnecessary high resource consumption [15], thereby wasting considerable human and financial resources. Another reason of high CS rates is that the perception of CS affords women an increased level of control over the birth, and they equate CS with safety and alleviation of fear. At present, more pregnant women consider that the CS delivery is safe because of the improvement of the surgical techniques, thereby leading them to select CS delivery mode. Questions about the economic implications of alternative modes of delivery have emerged as the rate of CS has continued to rise [4]. All the retrieved studies were reviewed independently by economists to decide whether they met criteria for relevance and quality. The quality of the economic evaluations and cost studies were assessed with criteria that were derived from the British Medical Journal checklist for economic evaluations [16].

In general, CS deliveries are obviously more expensive than vaginal deliveries [17]. In their recent review of the literature, Henderson determined what is known about the costs of CS deliveries compared with those of vaginal deliveries [16, 18]. According to a survey by WHO on delivery methods, the CS rates in China and other Asian countries were 46% and 27%, respectively, during 2007-2008. As expected, they determined that the literature consistently reported that cesarean deliveries were more expensive than vaginal deliveries. However, little is known about the costs of alternative modes of delivery. The increasing attention paid to the economic effect of delivery along with the increasing interest in the issue of CS is on demand. The economic assessments of obstetric interventions contribute important information in estimating the effect of obstetric practice on health service resources. With regard to the length of stay, Li et al. [19] determined that, after controlling age, race, or ethnicity, mothers with CS were 2.3 times more likely to require repeated hospitalization in the first postpartum 30 days than mothers without. The average initial hospital cost of a CS was 76% higher than the average cost of a vaginal birth. The length of stay of a CS was 77% longer than that of a vaginal delivery.

The majority of the previous studies on CS and vaginal deliveries paid attention to clinic delivery indications, but little attention was given to the association of costs and delivery modes. In this study, we aim to analyze two kinds of delivery modes, compare the costs of the two modes of delivery, and determine the influencing factors of the costs between the CS and vaginal deliveries in sample tertiary hospitals in Chongqing Municipality of China.

## 2. Material and Methods

The data of this study were drawn from obstetric medical cases of three tertiary hospitals in Chongqing Municipality of China during 2011–2013. All the information was collected from the National Health Statistical Information Report System (NHSIRS) of Chongqing Health Bureau Database. Chongqing Health Bureau authorized us to use the data.

A total of 18 tertiary hospitals in Chongqing Municipality of China existed, and each hospital had approximately 3000 to 3400 pregnant women each year. In this study, the total 30,168 obstetric medical cases included 10,897 vaginal delivery cases and 19,271 CS cases. All the obstetric medical cases were anonymized and without the private information of all the pregnant women (i.e., names, addresses, and contact ways). The obstetric medical cases were selected by excluding stillbirth and abortion. All of the patients in this study belonged to the normal childbearing age range of 15–60 years.

### 2.1. Statistical Analysis

All of the data were inputted into SPSS software (v.19.0, SPSS Inc., Chicago, IL, USA) for statistical analysis by all the participants in this study, and all the participants were trained before inputting all the data. Chi-square test was used to compare the distributions of vaginal delivery and CS under different indicators. Kruskal-Wallis test was adopted to analyze the differences of the delivery costs of the same delivery mode under different situations. However, Mann–Whitney test was used to test the differences of the delivery costs of different delivery modes under the same situation. We also adopted multiple linear regression and stepwise regression methods to select the main factors that influence delivery costs. In multiple linear regression method, the delivery costs of the total pregnant women (Model 1), the pregnant women with vaginal delivery (Model 2), and the pregnant women with CS (Model 3) were considered dependent variables. Meanwhile, the length of stay, areas, and modes of delivery, medical insurance modes, and maternal age were considered independent variables, whereas delivery expense was considered as a dependent variable.

## 3. Results

Different modes of delivery are described by adopting chi-square test as shown in Table 1. The total number of the samples is 30,168 including 10,897 cases of vaginal delivery and 19,271 of CS. The rates of vaginal delivery and CS were significantly different from 2011 to 2013, and all rates were growing annually (*P* = 0.000 < 0.005). The rate of both CS and vaginal delivery in the rural areas was larger than that in urban areas. Table 1 indicated that the number of the vaginal delivery cases in urban areas (48.36%) is less than that in rural areas (51.64%), whereas the number of CS cases in rural areas (53.81%) was more than that in urban areas (46.19%). Maternal age also had significant differences (*P* = 0.000 < 0.005). Based on maternal age, both vaginal and CS cases were mainly distributed in the age groups of 20–24 and 25–29. However, the rate of CS was considerably larger than vaginal delivery in the age group of 30 and older. Towards three basic medical insurances, Table 1 shows that the different medical insurances had significant differences (*P* = 0.000 < 0.005). More than 70% of pregnant women had insurance, and among them, more than 40% maternal women have NCMS, which covered the largest proportion among the three basic medical insurances. Table 1 shows that the length of stay also had significant differences among different periods (*P* = 0.000 < 0.005), and the majority of inpatients who undergo vaginal or CS stayed in the hospital for 4–8 days. However, 13.20% of women who undergo CS had to stay in the hospital for 9–12 days.

Table 2 shows that both costs of vaginal delivery and CS increase from 2011 to 2013 every year. In addition, both the costs of vaginal delivery and CS have significant differences (③ > ② > ①; *P* = 0.000 < 0.005). The costs of CS are higher than those of vaginal delivery (*P* = 0.000). The vaginal delivery costs of pregnant women from the urban areas are higher than those of pregnant women from the rural areas (*P* = 0.000). Similarly, the costs of CS of the pregnant women from the urban areas are higher than those of the pregnant women from the rural areas (*P* = 0.000). The result indicates that the delivery costs and maternal age have significant differences among the four age groups in the vaginal delivery group (*P* = 0.000). In vaginal delivery, the age groups of 20–24 and 25–29 spent more money than the age groups of 30–35 and above 35 among the four age groups. Significant differences also exist in the CS group (④ > ③ > ② > ①; *P* = 0.000) and indicated that the older the pregnant women are, the higher the CS costs are. However, the CS costs generally higher than vaginal delivery in any age group. Medical insurances also had significant differences. The results showed that the pregnant women with URBMI who chose vaginal delivery would spend the most among the four insurance groups. Given the CS costs, the pregnant women who had URBMI spent more than those who had UEBMI or NCMS. Nevertheless, the pregnant women who had URBMI and those who had other types of medical insurances had no significant differences. The results indicated that the longer the length of stay in the hospital, the higher the costs that would be spent. However, the costs of CS were more than those of vaginal delivery at the same length of stay.

Table 3 shows our analysis of influencing factors of delivery costs. In both Models 1 and 2, the delivery costs were affected by different factors (*P* < 0.01). The models also indicate that the delivery costs in urban areas were higher than those in rural areas. In general, the delivery costs were significantly affected by different modes of delivery. The costs of CS were higher than those of vaginal delivery (*P* < 0.01). Disparate payment modes of medical insurance also had significant differences according to the delivery costs (*P* < 0.05). Models 1 and 2 showed that pregnant women who had UEBMI and other kinds of medical insurance spent less in delivery costs than those who had URBMI. However, pregnant women with NCMS and those with URBMI had no significant difference in delivery cost. In Model 3, the pregnant women who had UEBMI spent less in delivery cost than those who had URBMI. However, their costs in NCMS and other insurance models were increased. In general, the delivery costs among the three regression models were significantly influenced by the maternal age and length of stay (*P* < 0.05); namely, the older the pregnant women are and the longer the length of stay is, the higher the delivery costs are. However, women who are younger than 30 years and those who adopted vaginal delivery do not obey previous findings.

## 4. Discussion

The rates of CS have increased beyond the recommended level of 15% in many countries all over the world and had been doubled in the last decade. The high CS rate is not only in high-income countries, but also in low-income countries, particularly for births in private hospitals. Table 1 shows that the number of pregnant women increased from 2011 to 2013 in Chongqing, China. Meanwhile, the CS rates and the vaginal delivery rates increased annually. In 2011 and 2012, the CS rates were higher than vaginal delivery rates. However, in 2013, the CS rate was lower than the vaginal delivery. The possible reasons include the improvement of people's quality of life, healthy diet, and accessibility of vaginal delivery clinical indicators. This study indicated that the number of the rural cases (48.36%) is less than that of the urban cases (51.64%) in vaginal delivery, whereas the number of rural cases (53.81%) is more than that of urban cases (46.19%) in CS. The possible reason is that many families, particularly the elderly, in rural areas want to choose a lucky day to give birth. Thus, they would give birth through the CS method. In addition, another reason was that rural women with complications (e.g., diabetes and hypertensions) in township hospitals or city hospitals will be referred to these tertiary hospitals that will lead to a higher CS rate. Some literature reviews have verified this kind of phenomenon [20, 21]. Some relative studies indicated that Chinese mothers prefer to choose a delivery date based on luck and belief, and delivering on a scheduled day via CS is easier than delivering an unplanned vaginal birth. The results show that the CS is concentrated on the age group of 20–24 and 25–29, which indicate that young pregnant women cannot endure the vaginal delivery pain [1]. The relative literature shows that some pregnant women worry about pain and vaginal tone after vaginal birth. These women also believe that CS is safer, faster, less painful, and maybe less likely to affect the quality of sexual life than vaginal birth [10]. In addition to the pain of pregnant women, many doctors worried about the medical dispute originated from the vaginal delivery risk, and the unreasonable requirement came from pregnant family members thereby increasing the CS rate in China. The results in Table 1 show that NCMS covers the largest proportion among the four medical insurances. NCMS was introduced in 2003 by the Chinese government. NCMS aims to decrease the medical care burden and to enhance people's quality of life. NCMS also focuses on people in rural areas. In this study, we selected maternal cases from three tertiary hospitals in Chongqing, in which more samples are from the rural areas than from the urban areas. In Chongqing, URBMI was integrated with NCMS as one scheme since 2010 [19].

However, the insurance policy will last for a long time from its introduction, pilot to comprehensive coverage. The data of our research came from 2011 to 2013; moreover, the coverage rate of medical insurance was 94.34% in 2015, and the less coverage rate was in 2013, which means the Urban-Rural Cooperative Medical Insurance was in the progress of implementation in 2013 [12]. In our study, the NCMS indicated the maternal women who came from rural areas with the Urban-Rural Cooperative Medical Insurance, while the URBMI indicated the maternal women who came from urban areas with Urban-Rural Cooperative Medical Insurance.

The results shown in Table 2 indicated that both delivery costs in urban areas are higher than those in rural areas. Except for the same basic delivery costs for staying at hospitals, the postnatal nutrition provided to pregnant women in urban areas is higher than that provided to pregnant women in rural areas. In addition, some unnecessary medical indications have added to the CS cases. Previous literature showed that the CS rate in China had increased nine times in the past five decades, and the urban rate was usually higher than rural rate in China [22].

Many factors influence the increase of CS rates in China [6], such as the increasing age of primiparous women, one-child policy, and financial benefits of opting for CS and professional interests. Among various reasons, the main reasons for the increasing CS rates in China should be the social and cultural factors instead of medical reasons [9]. Among Chinese populations, CS rates are affected by many nonmedical factors which include cultural issues, personal and social features of women, and health insurance coverage [18]. A further increase in the CS rate not only wastes the medical resources, but also causes more postoperative complications. According to Liu [23], the postoperative complication rate of CS is 17%, whereas that of the vaginal delivery is 10%. Hence, local and international scholars presented some effective measures to strengthen the intrapartum care and the antepartum health education, to increase obstetric staffing, and to improve the midwifery skills of young physicians [24].

In terms of delivery costs, particularly CS costs, one Canadian, five British, one Australian, one Italian, and two Swedish studies have cited CS costs. The costs ranged from £996 to £12,065 [25]. The results in Tables 2 and 3 show that the CS delivery costs increase along with the increase of pregnant women's age. The results also show that, among 10897 CS cases, 15.22% of CS delivery women were beyond the age of 30. Women with increased age have many severe and moderate complications and delivery difficulties. Meanwhile, increased medical staffing and increased length of stay lead to increased delivery costs. The CS rates of high-age pregnant women are high due to the fact that obstetric canal is not easy to expand and the uterine is weak [26, 27].

In terms of health care insurance, Tables 2 and 3 show that different sorts of medical insurances have significant differences. Health care insurance system may be a potential factor associated with the prevalence of CS. In China, the form of medical payment and the compensation difference between CS and vaginal delivery from insurance in China are important factors accounting for the increasing CS rate [28, 29]. In other countries, the health care system has been found to have perverse incentives for the increase of CS rates [5, 30]. Similarly, the results show that the costs caused by length of stay are different from those affected by the payment methods and distinct areas. Thus the hospital managers can take diagnosis-related groups (DRGs) to save costs and medical resources by standardizing the behavior of medical staff, implementing clinical pathway management, optimizing the delivery process, and shortening the length of stay.

The results show that the length of stay in CS is longer than that in vaginal delivery. The probable reason is the slow wound healing and long convalescence for CS patients. The additional costs of a CS are attributed to the additional staff inputs during the delivery itself and an extended inpatient stay [10, 31]. In this study, the average length of stay for vaginal delivery and CS is 5.41 and 6.86, respectively; however, these values are 1.74 and 3.01, respectively, in the United States in 1996 [32]. The long length of stay is closely related to the medical care level, custom, and postpartum family nursing. Hence, shortening the length of stay can control excessive growth of inpatient costs, improve resource utilization, and reduce medical cost [17]. In general, the costs of length of stay have been increasing from 2011 to 2013. The results revealed that pregnant age, modes of delivery, different areas, and length of stay which show significant differences influence the delivery costs. Thus, the payment standard of CS needs to be integrated with the features of inpatient costs themselves. In addition, the pregnant women are reasonably divided into groups, and the quota payment standards of each group are ensured.

### 4.1. Limitations

In view of the limitation of this study, we initially just limited the investigations on the data of the three tertiary hospitals in Chongqing from a view of hospital management, which is lack of clinical parameters. What is more, some factors about the complications occurring around time of childbirth were not be considered, which may be our next research step. More samples should come from secondary and primary hospitals in the future study to enhance the representativeness of this study. The clinical indications of pregnant women were not studied because many previous studies on the medical indications of CS and vaginal delivery have already existed. We studied the associated influencing factors of CS and vaginal delivery at the view of public health management. The importance of the clinical indications or parameters of delivery may be studied in the near future.

## 5. Conclusions

In this study, we realize that the CS rates in rural areas are larger than those in urban areas, and CS cases mainly emerge on the age groups of 20–24 and 25–29. NCMS covers the largest proportion of the basic medical insurances. The longer the length of stay is, the more expensive the delivery costs are.

CS rates in China vary remarkably by regions [20, 33] and among cities and counties [32]. Some of the cesarean indications in China are unique, and the increase in cesarean delivery rate tends to be multifactorial. The previous studies in China determined that perinatal mortality decreased as the rate of CS increased until this rate reached approximately 20%–25%, after which perinatal mortality rate leveled off [31, 34–36]. The CS rates in China have been increasing [37–39].

At present, the increased CS rates and medical costs need to be noticed. However, the payment system reform of medical costs is crucial. The medical insurance institution can adopt capitation or DRGs to ensure a payment standard based on the objective indications to strengthen doctors' comprehensive skills and to realize delivery indications and prenatal health education. Meanwhile, hospitals can control medical costs, shorten the average length of stay, and accelerate the bed turnover on the premise of guaranteeing medical quality. On the contrary, given the increase in CS rates, pregnant women should pay attention to healthy diets and perform exercises to extend the rates of vaginal delivery.

## Figures and Tables

**Table 1:** Distribution of different modes of delivery.

Category	Sum	Vaginal delivery	CS	*χ* ^2^	*P*
*Sum*	*N* = 30168	*N* = 10897	*N* = 19271		
*Year*					
2011	7713 (25.57%)	2391 (21.94%)	5322 (27.62%)	213.37	0.000
2012	10462 (34.68%)	3612 (33.15%)	6850 (35.54%)		
2013	11993 (39.75%)	4894 (44.91%)	7099 (36.84%)		
*Areas*					
Rural	15640 (51.84%)	5270 (48.36%)	10370 (53.81%)	82.80	0.000
Urban	14528 (48.16%)	5627 (51.64%)	8901 (46.19%)		
*Age*					
20–24	10101 (33.48%)	4169 (38.25%)	5932 (30.78%)	743.89	0.000
25–29	12869 (42.66%)	5070 (46.53%)	7799 (40.47%)		
30–34	5414 (17.95%)	1349 (12.38%)	4065 (21.10%)		
≥35	1784 (5.91%)	309 (2.84%)	1475 (7.65%)		
*Medical insurance*					
URBMI	3869 (12.83%)	1546 (14.19%)	2323 (12.05%)	88.14	0.000
UEBMI	2665 (8.83%)	1050 (9.64%)	1615 (8.38%)		
NCMS	15432 (51.15%)	5062 (46.45%)	10370 (53.82%)		
Others	8202 (27.19%)	3239 (29.72%)	4963 (25.75%)		
*Length of stay*					
1–3	819 (2.72%)	771 (7.08%)	48 (0.25%)	1767.99	0.000
4–8	25335 (83.98%)	9397 (86.23%)	15938 (82.70%)		
9–12	3129 (10.37%)	585 (5.37%)	2544 (13.20%)		
≥13	885 (2.93%)	144 (1.32%)	741 (3.85%)		

**Table 2:** Comparison of delivery costs in different modes of delivery.

Category	Vaginal delivery		Caesarean		*P*, sig.^b^
	Median	*Q*25–*Q*75	Median	*Q*25–*Q*75	
*Year*					
① 2011	2895.60	2509.63–3353.24	4448.91	3957.19–5071.10	0.000
② 2012	3003.17	2692.46–3452.03	4591.63	4095.70–5216.80	0.000
③ 2013	3275.39	2803.60–3829.90	4697.72	4198.78–5396.37	0.000
*P*, sig.^a^(LSD)	0.000	③ > ② > ①	0.000	③ > ② > ①	
*Areas*					
① Rural	3032.79	2674.14–3547.91	4569.58	4062.51–5192.86	0.000
② Urban	3139.93	2711.30–3680.50	4614.60	4121.34–5316.30	0.000
*P*, sig.^b^	0.000	② > ①	0.000	② > ①	
*Age*					
① 20–24	3086.91	2685.36–3589.13	4405.54	3956.22–5006.83	0.000
② 25–29	3143.52	2735.20–3676.12	4503.84	4034.63–5177.57	0.000
③ 30–34	2954.90	2598.99–3526.20	4834.24	4354.50–5444.77	0.000
④ ≥35	28720.00	2433.71–3334.40	5038.94	4476.30–5848.47	0.000
*P*, sig.^a^(LSD)	0.000	① > ③ > ④, ② > ③ > ④	0.000	④ > ③ > ② > ①	
*Medical insurance*					
① URBMI	3260.22	2800.00–3835.41	4655.40	4154.70–5408.00	0.000
② UEBMI	3090.73	2670.79–3568.81	4460.04	4020.14–5165.42	0.000
③ NCMS	3032.79	2674.14–3547.91	4569.58	4062.51–5192.86	0.000
④ Others	3098.30	2687.00–3652.60	4650.40	4143.00–5332.60	0.000
*P*, sig.^a^(LSD)	0.000	① > ②, ① > ④ > ③	0.000	① > ②, ① > ③, ② < ③ < ④	
*Length of stay*					
① 1–3	2426.13	2194.50–2755.02	3896.94	3533.97–4509.60	0.000
② 4–8	3083.90	2718.60–3550.92	4426.89	4005.25–4948.82	0.000
③ 9–12	4185.90	3761.20–4681.40	5482.37	4976.68–6094.18	0.000
④ ≥13	5116.57	4522.92–5770.90	6493.67	5931.08–7268.48	0.000
*P*, sig.^a^(LSD)	0.000	① < ② < ③ < ④	0.000	① < ② < ③ < ④	

^a^Based on Kruskal-Wallis test. Compare differences of delivery costs within the same mode of delivery which were under different indicators.

^b^Based on Mann–Whitney test. Compare differences of delivery costs within the two kinds of different modes of delivery which were under the same indicators.

**Table 3:** Impact factors of pregnant costs.

Indications	B	SE	Beta	*t*	*P*
*Model 1 (sum)*					
*Constant*	1027.163	31.926		32.173	0.000
*Areas*					
Rural (control group)					
Urban	66.572	13.676	0.028	4.868	0.000
*Modes of delivery*					
Vaginal delivery (control group)					
Caesarean	1358.515	9.317	0.558	145.818	0.000
*Medical insurance*					
URBMI (control group)					
UEBMI	−138.224	18.698	−0.034	−7.392	0.000
Others	−34.574	14.608	−0.013	−2.367	0.018
*Age*					
① 20–24 (control group)					
② 25–29	60.684	10.275	0.026	5.906	0.000
③ 30–34	258.513	12.718	0.085	20.326	0.000
④ ≥35	425.021	19.381	0.086	21.930	0.000
*Length of stay*					
① 1–3 days (control group)					
② 4–8	753.831	26.934	0.237	27.988	0.000
③ 9–12	1806.093	30.024	0.471	60.156	0.000
④ ≥13	2789.325	36.760	0.403	75.879	0.000

*Model 2 (vaginal delivery)*					
*Constant*	2491.676	34.127		73.012	0.000
*Areas*					
Rural (control group)					
Urban	152.519	19.108	0.102	7.982	0.000
*Medical insurance*					
URBMI (control group)					
UEBMI	−175.545	25.644	−0.069	−6.846	0.000
Others	−120.589	20.142	−0.072	−5.987	0.000
*Age*					
① 20–24 (control group)					
② 25–29	20.535	14.050	0.014	1.462	0.144
③ 30–34	−91.254	20.386	−0.040	−4.476	0.000
④ ≥35	−181.580	38.039	−0.040	−4.773	0.000
*Length of stay*					
① 1–3 days (control group)					
② 4–8	692.059	24.213	0.318	28.582	0.000
③ 9–12	1745.953	35.277	0.524	49.493	0.000
④ ≥13	2682.909	58.287	0.408	46.029	0.000

*Model 3 (CS)*					
*Constant*	3767.145	115.271		32.681	0.000
*Medical insurance*					
URBMI (control group )					
UEBMI	−109.880	25.424	−0.032	−4.322	0.000
Others	24.872	19.800	0.011	1.256	0.209
*Age*					
① 20–24 (control group)					
② 25–29	88.119	14.011	0.045	6.289	0.000
③ 30–34	399.572	16.099	0.170	24.82	0.000
④ ≥35	586.365	23.101	0.163	25.382	0.000
*Length of stay*					
① 1–3 days (control group)					
② 4–8	685.914	113.452	0.271	6.046	0.000
③ 9–12	1741.452	114.351	0.616	15.229	0.000
④ ≥13	2734.644	116.880	0.549	23.397	0.000
